# Functional characteristics and subjective disease perception in patients with COVID-19 two months after hospital discharge

**DOI:** 10.3389/fresc.2023.1209900

**Published:** 2023-07-21

**Authors:** Kaja Teraž, Boštjan Šimunič, Manca Peskar, Uros Marusic, Saša Pišot, Luka Šlosar, Malden Gasparini, Rado Pišot

**Affiliations:** ^1^ Institute for Kinesiology Research, Science and Research Centre Koper, Koper, Slovenia; ^2^ Faculty of Sport, University of Ljubljana, Ljubljana, Slovenia; ^3^ Biological Psychology and Neuroergonomics, Department of Psychology and Ergonomics, Faculty V: Mechanical Engineering and Transport Systems, Technische Universität Berlin, Berlin, Germany; ^4^ Department of Health Sciences, Alma Mater Europaea – ECM, Maribor, Slovenia; ^5^ Department of General Surgery, General Hospital Izola, Izola, Slovenia

**Keywords:** coronavirus, recovery, functional improvement, hospital stay, health perception

## Abstract

**Introduction:**

Although early inpatient and post-hospital rehabilitation is recognized as necessary, not all COVID-19 patients have access to rehabilitation. There are no published reports in the literature that investigate the outcomes of patients who do not receive rehabilitation after COVID-19. Our aim was to evaluate possible improvements in determinate functional and psychological parameters in COVID-19 patients two months after their hospital discharge.

**Methods:**

On both time points various motor, cognitive, and clinical measurements such as body composition, tensiomyography, blood pressure, spirometry, grip strength test, Timed Up and Go test, gait speed, 30-second chair-stand test, and Montreal Cognitive Assessment, were performed. Additionally, questionnaires such as the SARC-CalF test, Edmonton frail scale, International Physical Activity questionnaire andThe Mediterranean Lifestyle index were conducted to assess lifestyle characteristics.

**Results:**

A total of 39 patients (87.2% male; mean age of 59.1 ± 10.3 years), who were hospitalized due to COVID-19 at the Izola General Hospital (IGH), Slovenia between December 2020 and April 2021, were included. Patients were assessed at two time points (T_1_ and T_2_): T_1_ was taken after receiving a negative COVID-19 test and T_2_ was taken two months after T_1_. After two months of self-rehabilitation, we have detected a BMI increase (*p* < .001), fat free mass increase (*p* < .001), better Edmonton frail scale (*p* < .001), SARC-CalF score (*p* = .014) and MoCA score (*p* = .014). There were no detected changes in lifestyle habits nor in physical performance tests.

**Discussion:**

It is already known that COVID-19 has long-term negative consequences regardless of the stage of the disease. Our findings support the notion that patients cannot fully regain all their functions within a two-month period without receiving structured or supervised rehabilitation. Therefore, it is crucial to offer patients comprehensive and structured rehabilitation that incorporates clinical, cognitive, and motor exercises.

## 1. Introduction

In March 2020, the World Health Organization declared coronavirus disease 2019 (COVID-19) a pandemic (1). The first case of COVID-19 in Slovenia was reported on 04/03/2020 (2). By 20/10/2022, more than 620 million confirmed cases had been reported worldwide, with more than 5.5 million deaths (3). At that time, there were 1.2 million confirmed cases in Slovenia, including 8,368 deaths (3).

The severity of COVID-19 disease ranges from no symptoms to mild flu-like symptoms to severe pneumonia and acute respiratory distress syndrome (ARDS) (4–6). Previous studies have described details at admission, such as clinical characteristics (e.g., fever, acute respiratory distress syndrome, abnormal chest radiograph, shortness of breath, fever) (7–10), demographics (a higher proportion of infected patients comprised men, medical staff, and hospitalized patients) (5, 10–12), comorbidities (hypertension, obesity, and diabetes (5, 8, 9), and laboratory parameters (e.g., higher plasma levels of IL2, IL7, TNFα, etc (9). It is already known that the disease can cause various types of damage to multiple organs, particularly the brain (13). The inflammatory response that is triggered by the infection can lead to long-term cognitive decline, psychological distress, and musculoskeletal issues (14–16). Moreover, patients who have survived severe COVID-19 disease exhibit impaired balance and strength (17). Muscle contractile properties, assessed by tensiomyography (TMG), were followed in Slovenian football players after approximately 2 months of COVID-19 lockdown (18). When compared post- to pre-, they reported a 6% and 50% increase in contraction time in the vastus lateralis and biceps femoris, respectively. Furthermore, this shift was not related to jump performance but was associated with increased injury incidence. However, TMG was not used in participants after COVID-19 hospitalization. All of these consequences of COVID-19 also lead to the development of frailty, sarcopenia, and decreased quality of life, which can last for up to a year after the disease has subsided (19).

The different functional and medical conditions of patients before the onset of the disease, combined with the different recovery rates after the disease, led to the development of several guidelines and recommendations for the rehabilitation of patients after COVID-19 (20). Although it is already well known that early inpatient and post-hospital rehabilitation is necessary, regular, systematic, and guided rehabilitation after COVID-19 is not available to all patients. We did not find any reports in the literature examining what happens to patients who are not admitted to rehabilitation after COVID-19. In addition to the guidelines for professional rehabilitation, many organizations have published guidelines to promote self-rehabilitation after COVID-19. The World Health Organization encouraged individuals to be proactive by publishing guidelines for self-rehabilitation after COVID-19 (21), although the literature supporting this self-management practice among individuals rehabilitating after COVID-19 is sparse (20). Therefore, we were interested in investigating possible changes in some physiological and psychological parameters in COVID-19 patients who were not enrolled in specific rehabilitation programs after hospital discharge. However, all patients received a general booklet with recommendations and instructions for regular physical activity, which was not specifically designed for patients with COVID-19. It was assumed that most of the measured parameters would improve two months after hospital discharge, despite the fact that the patients were not enrolled in a specific rehabilitation program.

## 2. Methods

We conducted a single-center prospective cohort study by enrolling 43 consecutively hospitalized patients that were admitted to the General Hospital Izola due to a complicated COVID-19 disease. Functional and clinical characteristics at hospital discharge (T_1_) and 2-month follow-up (T_2_) were collected until 17/05/2021.

### 2.1. Participants

Initially, 43 participants were included in the study, but four of them did not appear for the second measurement, leaving a total of 39 patients tested at both time points (T_1_ and T_2_). Participants were recruited from the pool of patients hospitalized at the Department of Internal Medicine of the Izola General Hospital, Slovenia, due to COVID-19 and its complications. Inclusion criteria for participation in the study were: age ≥18 years, signed informed consent, and hospitalization for COVID-19 disease [confirmed with a positive polymerase chain reaction (PCR) nasal swab test for SARS-CoV-2 virus]. Exclusion criteria were a positive PCR test for the SARS-CoV-2 virus at the time of hospital discharge and severe medical conditions (musculoskeletal, cardiovascular, pulmonary, and neurological) that prevented patients from performing all motor and cognitive tests. All patients were visited by a dedicated physician during their hospitalization, and the study protocol was discussed in detail with them. If the participant agreed to participate in the study, written informed consent was obtained prior to each examination. The clinical trial protocol was registered on ClinicalTrials.gov under the identifier number NCT04860206. All procedures were performed in accordance with the Declaration of Helsinki, and the study was ethically approved by the institutional ethical board of the Izola General Hospital (application number: 1/21).

Patients participated in T_1_ measurements on the condition of a negative COVID-19 test, which was performed when one of the following conditions was met: on the day of hospital discharge or the 10th day after confirmation of the disease if discharged from the hospital earlier. The time frame for recruiting patients for T_1_ measurements was between 01/01/2021 and 18/03/2021. After the T_1_ assessment, the subject received a “Stay Active” brochure (22) with general information on the beneficial effects of physical activity and some comprehensive explanation of how different exercises could be performed at home during the COVID-19 pandemic. The T_2_ assessment was conducted 2 months after the T_1_ assessment (the time frame for T_2_ measurements was between 11/03/2021 and 19/05/2021). All assessments were performed at the Cardiac Rehabilitation Center of Izola General Hospital during the morning hours.

### 2.2. Measurements

All assessments at T_1_ and T_2_ were performed by a trained researcher in the same room and using the same equipment. Measurements were taken in this order:

Body mass (kg) and height (m) were measured with a Libela personal scale with a mounted stadiometer (Libela-Elsi Ltd., Slovenia), and the results were rounded to the nearest 0.1 kg and 0.5 cm, respectively. Body composition was measured using the tetrapolar bioimpedance device BIA 101 Anniversary (Akern-Srl, Florence, Italy) after the participants were in the supine position for 30 min. The proportions of fat mass (FM in %) and muscle mass (MM in %) were recorded from the assessment.

Tensiomyography measurements were performed in three muscles of the right leg: the vastus lateralis (VL), the biceps femoris (BF), and the gastrocnemius medialis (GM). All measurements were made during electrically evoked maximal isometric twitch contractions. For the VL, participants were in the supine position with the knee angle at 30° of flexion (where 0° represents a fully extended knee joint). For the BF, they were in the prone position with the knee at 5° of flexion, and for the GM, they were prone with the ankle in a neutral position, as previously reported (23–25). Foam pads were used for joint support. A single 1-ms maximal monophasic electrical impulse was used to elicit a twitch, which caused the muscle belly to oscillate and enlarge. These oscillations were recorded using a sensitive digital displacement sensor (TMG-BMC Ltd., Slovenia) that was placed on the surface of the skin over the mid-belly of the muscle of interest. If needed, the measurement point and electrode positions were adjusted to obtain the maximum amplitude (Dm) of the muscle belly response. Initially, the stimulation amplitude was set just above the threshold and then gradually increased until the Dm of the radial twitch displacement did not increase any further. From two maximal twitch responses, a contraction time (Tc), a delay time (Td), and a transversal velocity (Vc) were calculated, and the average was used for further analysis. Td was defined as the time from the electrical impulse to 10% of Dm. Tc was defined as the time for the amplitude to increase from 10% to 90% of Dm (25). Vc was calculated as the ratio between Dm and the sum of Td and Tc (26).

During the tensiomyography assessment, participants were interviewed to elucidate possible unhealthy lifestyle behaviors and to estimate their risk for sarcopenia and frailty conditions. For this purpose, we used the Slovenian version of the SARC-CalF questionnaire (27), the Mediterranean Lifestyle Index (MEDLIFE) (28), and the Edmonton frail scale questionnaire (29).

The SARC-CalF questionnaire is a screening tool for sarcopenia in older adults. It addresses five domains: strength, assistance in walking, rising from a chair, climbing stairs, and falling (30), and also includes a calf circumference measurement (31). Each answer is scored from 0 to 2 points according to the reported difficulty in performing the task in question. For calf circumference, zero represents normal muscle mass and 10 represents very low muscle mass. The sum of the points gives a score that can range from 0 to 20, with zero indicating the best result and 20 the worst. Individuals with a SARC-CalF score ≥11 are at increased risk for sarcopenia.

The Mediterranean Lifestyle index (MEDLIFE) is a tool that measures the adherence of the individual to the principles of the Mediterranean lifestyle (28). A total of 28 items are divided into three blocks of questions (food consumption frequency, Mediterranean dietary habits, and social habits). For each item, 1 point is awarded (28 points in total) if the answer meets certain criteria. The final score of the MEDLIFE index ranges from 0 (the worst) to 28 (the best).

The Edmonton frail scale (EFS) is a brief, valid, and reliable tool for assessing frailty. It can be administered by researchers without special training in geriatric medicine. The EFS assesses nine subscales: (1) cognition; (2) general health status; (3) functional independence; (4) social support; (5) medication use; (6) nutrition; (7) mood; (8) continence; and (9) functional performance. The EFS scores range from zero to 17. Severe frailty is defined as a score of 12–17, apparent frailty as a score of 6–11, and absence of frailty as a score of 5 or less (29).

The level of physical activity was determined with the Slovenian translation of the short version of the International Physical Activity Questionnaire (IPAQ), which was assessed at T_2_ (32, 33). A total of seven items assess the frequency (days per week), duration (time per day), and intensity (light, moderate, or vigorous) of PA, which was performed during the previous week.

### 2.3. Clinical tests

Blood pressure and pulse wave velocity were measured using a Vicorder (Vascular Model, SMT Medical Ltd., Germany). Prior to measurement, participants were placed in the supine position in a quiet room, with the head elevated to approximately 15°, so that the skin and muscles over the carotid artery were relaxed, but not too tense. Pulse wave velocity was measured with a cuff placed over the right carotid artery and the right thigh. The length between the common carotid artery (CCA) and the superficial femoral artery (SFA) was measured between the suprasternal notch and the midpoint of the thigh cuff. Measurements were taken until pressure waveforms across the CCA and SFA were clear and reproducible. During the FU, the same distance between the measurement sites was used. Blood pressure was measured in the sitting position with the cuff placed on the left upper arm.

Spirometry was performed with the participant comfortably seated in a chair. A clip was used to close the nostrils. Participants were instructed to breathe normally for 10 s and then to inhale as deeply as possible and exhale as quickly as possible into a tube (microQuark, COSMED Ltd., Italy). The best forced vital capacity (FVC) and forced expiratory volume measurement in one second (FEV1) of the three tests performed were used. An FEV1/FVC Tiffeneau-Pinelli index was calculated automatically (34).

### 2.4. Physical performance measurements

The grip strength test (35) was performed using an analog hydraulic handheld dynamometer (Jamar Dynamometer, Sammons Preston, USA). During the measurement, participants sat on a chair with no armrests. The dominant upper arm was parallel to the torso, while the elbow was positioned at 90° flexion. After two test attempts, participants performed three consecutive handgrips with a 1-minute rest in between. The best result was used for further analysis.

A “Timed Up and Go” test (36) was used to assess mobility at a distance of three meters. Participants wore their regular walking shoes. The test started with the participant sitting on a 46-cm-high chair, then standing up, walking around a marked bar, returning to the chair, and sitting on it. Each participant completed one test trial and two trials without walking aids. The trial with the shortest completion time was used for further analysis.

Gait speed was evaluated (37) at the self-selected speed and the fastest speed over a distance of 4 meters using timed gates (Beam Trainer timing system, Seedgrov d.o.o., Ljubljana, Slovenia). Two meters were provided before and after the timed distance for acceleration and deceleration. Each gait modality (self-selected speed and fast speed) was assessed twice, and the best result was used for further analysis.

A 30-second chair-stand test was used to assess the number of stands a participant could complete in 30 s (38). Participants initially sat on a 46-cm-high chair without armrests. They placed their hands on the opposite shoulder, crossed at the wrists. Their feet were flat on the floor, and their backs were straight. They rose to a full standing position, then sat back down again and repeated this for 30 s. Only completed repetitions were counted.

### 2.5. Cognitive tests

The Montreal Cognitive Assessment (MoCA) was used to evaluate the general level of cognitive functioning and screen for cognitive impairment (39). The MoCA test addresses several cognitive domains, namely visuospatial ability, short-term memory, executive function, attention, concentration, working memory, language, and orientation to time and place. The final score ranges from 0 to 30 points, with values ≥26 indicating no cognitive impairment.

### 2.6. Qualitative assessments

A structured interview was used to assess the self-perceived response to the COVID-19 experience in relation to functional, mental, and mood states. The structured interview was used to capture qualitative data. Patients were interviewed twice, at the first measurement and then at the second measurement after self-rehabilitation at an interval of 8 weeks. The interview was conducted after the functional tests between the patient and the researcher, who took notes on the answers.

The first structured interview included questions about the subjective experience of being hospitalized for COVID-19, i.e., what was most distressing for the respondent besides the physical problems: how did they help themselves or what helped them, and what did they miss most? They also rated their physical and mental states (in percentages) after the infection, assuming that their pre-infection state was rated as 100%. At the end of the interview, the patients were given a form to monitor their symptoms or changes in well-being and were asked to bring it to their post-rehabilitation follow-up appointment. Patients also received a “Stay Home—Be Active” manual with instructions on how to begin the exercise program designed for patients with chronic lung disease.

At T_2_, patients were asked to compare their physical and mental state and mood again, assuming that their pre-infection state was rated as 100%. They were also asked to evaluate in more detail what they found most difficult in coping with the physical and mental stress, whether they were frustrated, what thoughts came to mind during the recovery period, how they helped themselves if necessary, and what they missed during the recovery period or how it could have been more successful. They also assessed their perception of what it would take to reach 100% physical, mental, and emotional health and when they expected to achieve it. Finally, they were asked to hand over the symptom monitoring form they received at the first measurement, and if they did not bring it with them, they were interviewed by the researcher to collect the data. They were also asked if they had followed the advice in the manual and if they had exercised according to its instructions.

### 2.7. Statistics

**Figure 1:**
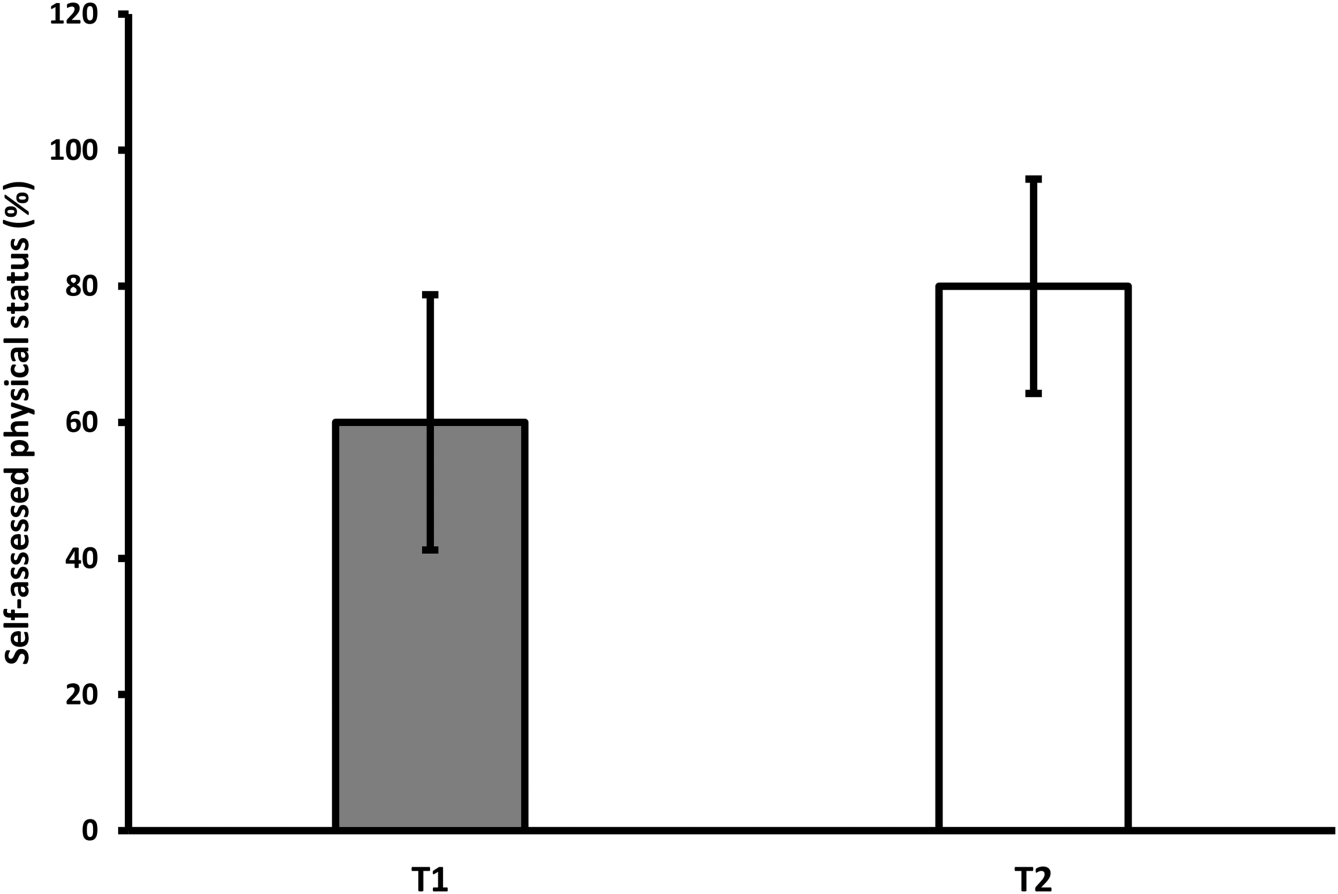
Average subjective assessment of physical status at the first and second measurements. *T_1_-first measurement; T_2_-second measurement.

**Figure 2:**
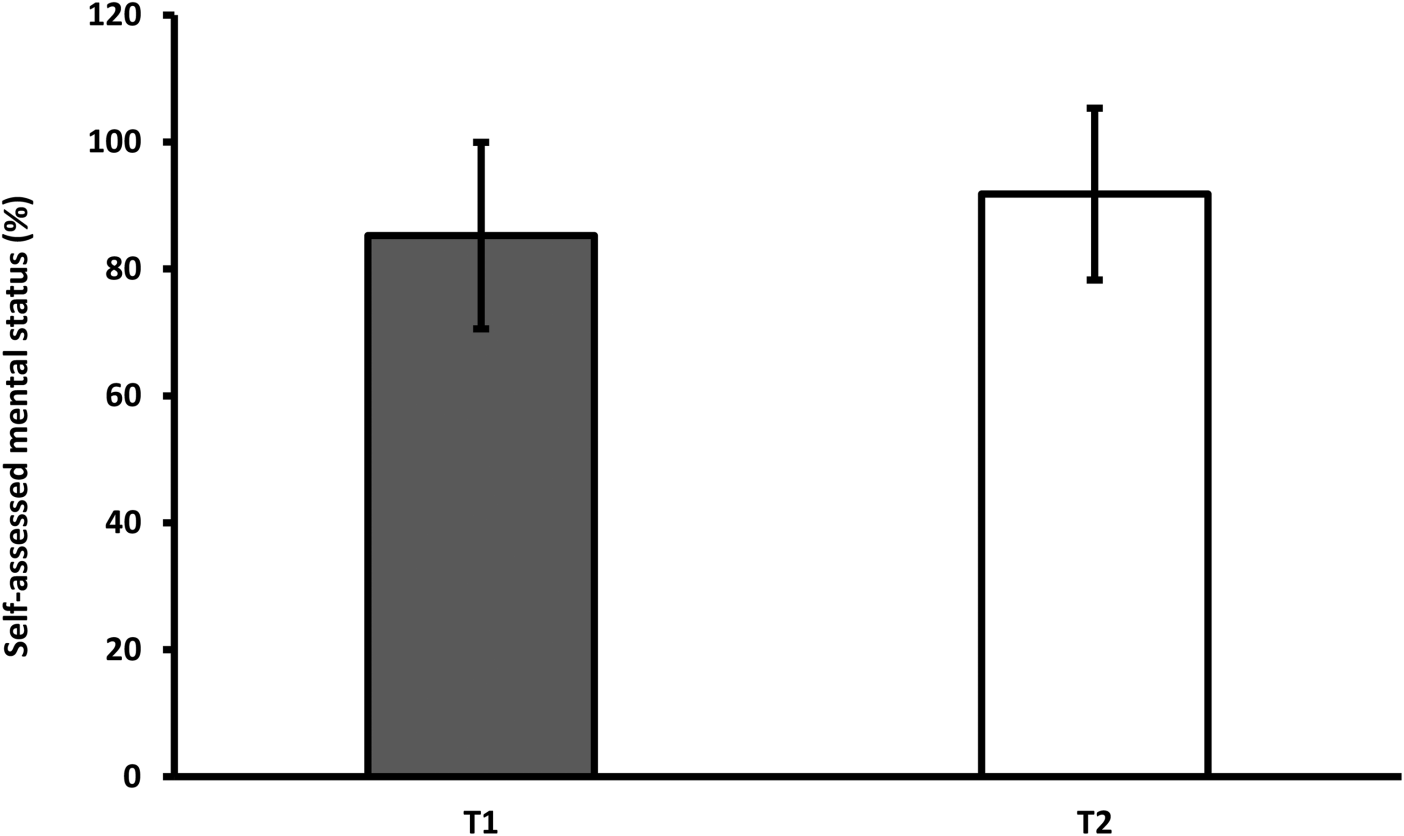
Average subjective assessment of mental status at the first and second measurements. *T_1_-first measurement; T_2_-second measurement.

**Figure 3:**
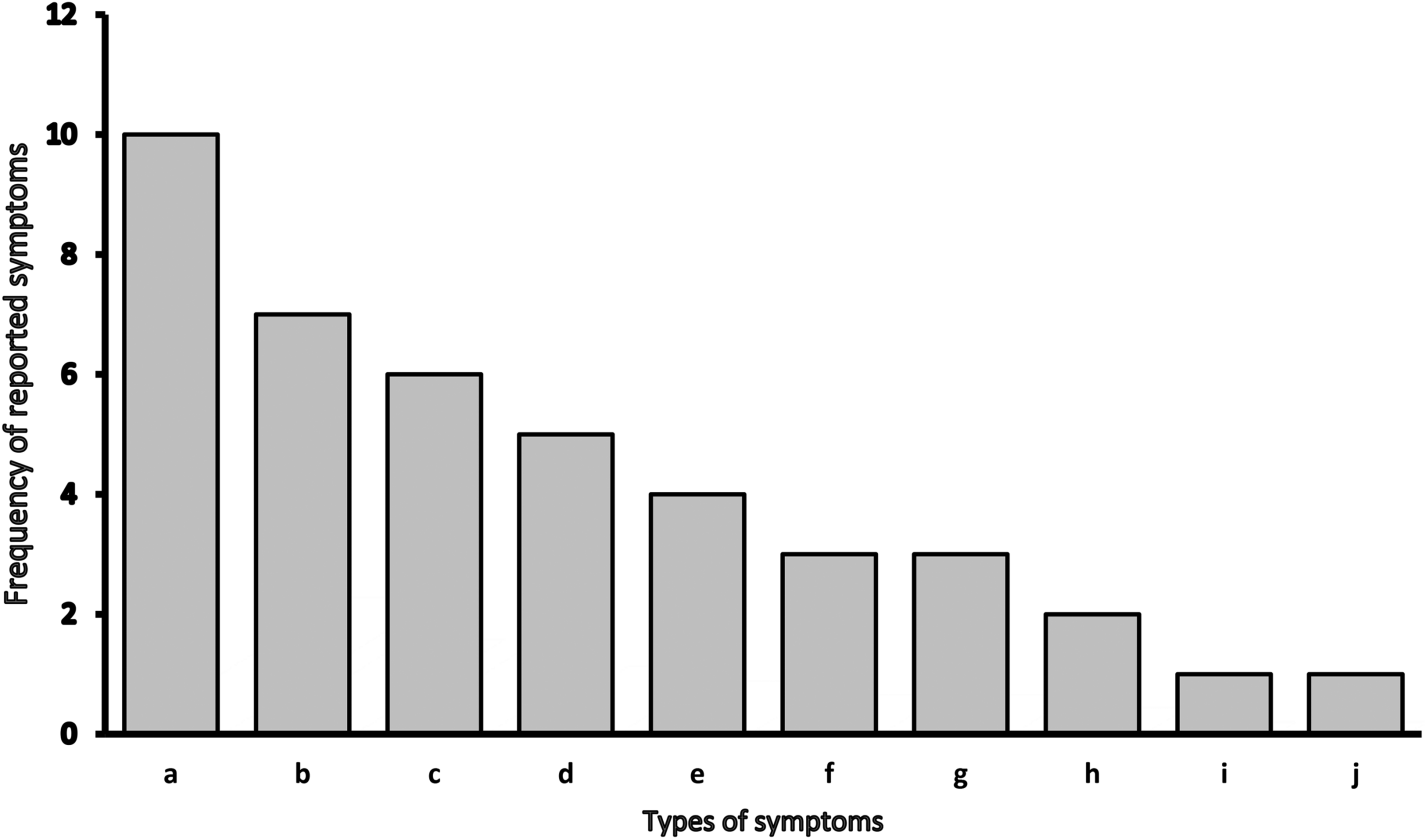
Types of symptoms and changes in well-being during self-rehabilitation. *a - joint pain; b - muscle pain, cough; c - irritability, sadness; d - shortness of breath, difficulty sleeping, e - severe headache, nausea; f - indigestion (constipation), poor sense of smell, poor taste; g – no listed symptoms; h - anxiety; i – fever, low appetite, increase appetite; j – other: tingling, impaired vision, fluctuating blood pressure.

We used G-Power (40) to determine the required sample size. Considering a two-sided *α*-value of 0.05 and a *β*-value of 0.20, and a functional decline of less than −11% (indicating an effect size greater than 0.9), we calculated that a sample size of 13 participants would be sufficient. All parameters are presented as the mean and standard deviation (SD). The normal distribution was confirmed by visual inspection using histograms and Q-Q plots, and analytically using the Shapiro-Wilk test. Parameters of the baseline (T_1_) and follow-up (T_2_) samples were assessed by a paired samples *t*-test. All statistical analyses were performed using IBM SPSS Statistics 22 (SAS Institute, Cary, NC, USA), with a significance level of *p* < .05. Figures 1–3 have been prepared using Microsoft Office Excel (Microsoft Corporation, Washington, USA).

#### 2.7.1 Qualitative analysis

For research purposes, we developed an index of subjective evaluation (of physical fitness, mental well-being, and mood) by using index equations commonly used in monitoring economic phenomena (measuring price changes) to show the extent of relative changes in a phenomenon over time. Interpretative phenomenological analysis (IPA) (41) was used to explore participants’ views of their own experiences of hospitalization due to the COVID-19 infection. This type of individual personal perception of the event as a phenomenon gave us the opportunity to produce an objective statement of such an event as the COVID-19 infection. Therefore, IPA can help us to illuminate the subjective perceptual processes and understand different responses to the same diagnosis.

Subjective assessment of physical and mental level was analyzed by index (I), in percentage for the period after hospitalization (T_1_) and after self-rehabilitation (T_2_) in relation to the pre-infection state, considered 100%. The difference between the two assessments was described by the index using the following equation:

Equation 1:

$$I=\frac{\mathrm{Yr}}{\mathrm{Yh}}*100$$

*I*—index of subjective assessment.
Yh—subjective assessment of physical or mental state after hospitalization in %.
Yr—subjective assessment of physical or mental state after self-rehabilitation in %.

An index above 100 means that the subjective state after self-rehabilitation (Yr) is higher than after hospitalization (Yh), so self-rehabilitation shows positive trends, while a value below 100 tells us that the post-self-rehabilitation state was estimated to be lower than after hospitalization. A value around 100 shows no or little change in the estimated state between Yh and Yr. The difference is expressed in percentage points.

Additionally, descriptive analysis was used to report the most common symptoms that patients had, which were assessed by the Monitoring symptoms form. Such analysis gave us an answer to “what happened” regarding the post-COVID-19 symptoms in the period of 6-weeks of self-rehabilitation.

## 3. Results

In the study analysis, we included 39 participants with a mean age of 59.1 ± 10.3 years (12.8% women) (Table 1).

**Table 1:** Basic data of the study participants at the baseline assessment.

	All
*N*	39
Age (years), mean ± SD	59.1 ± 10.3
Height (cm)	175.9 ± 8.7
Body mass (kg)	98.9 ± 16.6
Body Mass Index (kg/m^2^)	31.5 ± 4.7
Fat mass (%)	30.0 ± 10.5
Fat-free mass (kg)	66.9 ± 10.4
Number of hospital days	7.5 ± 6.0
Physical activity (MET min/day)	312.8 ± 396.8
Sedentary behavior (min/day)	323.5 ± 181.6
Smoking status, *n* (%)	
Current smoker	1 (2.6)
Former smoker	6 (15.4)
Comorbidities	
Hypertension	17 (43.6)
Coronary heart disease	3 (7.7)
Heart failure	2 (5.1)
Atrial fibrillation	2 (5.1)
Asthma	2 (5.1)
COPD	1 (2.6)
Diabetes I	0
Diabetes II	8 (20.5)

COPD, Chronic obstructive pulmonary disease.

Clinical, cognitive, and physical performance findings are summarized in Table 2. Compared to T_1_, BMI increased by 2.9% at T_2_ (*p* < .001, Cohen's *d* = 0.19), paralleled by a 4.8% increase in fat-free mass (*p* < .001, Cohen's *d* = 0.31). There were no changes in other body characteristics or physical or clinical tests. The only improvements we observed were in the Edmonton total symptom score (*p* < .001, Cohen's *d* = 0.64), the SARC-CalF score (*p* = .014, Cohen's *d* = 0.50), and the MoCA score (*p* = .014, Cohen's *d* = 0.35).

**Table 2:** Clinical, functional, and cognitive results at hospital discharge (T_1_) and 2-month follow-up (T_2_).

	T_1_	T_2_	
	Mean (SD)	Mean (SD)	*p*_TIME_ (*η*^2^)
Body mass (kg)	98.9 (16.6)	98.7 (16.3)	0.774
Body Mass Index (kg/m^2^)	31.5 (4.7)	32.4 (5.0)	<.001 (0.19)
Fat mass (%)	30.0 (10.5)	29.5 (11.5)	0.529
Fat-free mass (kg)	66.9 (10.4)	70.1 (11.5)	<.001 (0.31)
30-sec Sit-to-stand test (n)	14.7 (4.7)	15.4 (4.1)	0.304
Timed Up and Go (s)	6.4 (1.6)	6.3 (1.4)	0.767
Self-selected gait speed (m/s)	1.31 (0.24)	1.33 (0.25)	0.443
Fast-paced gait speed (m/s)	1.82 (0.32)	1.80 (0.28)	0.610
Grip strength (kg)	41.6 (11.7)	41.8 (13.0)	0.732
Systolic blood pressure (mmHg)	143.8 (17.3)	148.6 (19.5)	0.263
Diastolic blood pressure (mmHg)	76.4 (11.1)	79.4 (11.1)	0.264
FEV1/FVC	0.80 (0.08)	0.8 (0.07)	0.424
MEDLIFE index	14.10 (4.1)	13.23 (4.3)	0.170
EDMONTON Scale	3.41 (2.5)	1.82 (2.2)	<.001 (0.64)
SARC-CalF Score	1.45 (1.5)	0.7 (1.0)	0.014 (0.50)
MoCA Score	24.3 (2.9)	25.3 (3.2)	0.014 (0.35)

FEV1, forced expiratory volume in one second; FVC, forced vital capacity; MEDLIFE, Mediterranean lifestyle; MoCA, Montreal Cognitive Assessment score.

Tensiomyographic data (Table 3) showed a decrease in Tc in all three muscles at T_2_ when compared to T_1_. Similarly, Td decreased in VL and GM, whereas it was almost significant in BF. Since Dm was unchanged, Vc increased, but only in BF.

**Table 3:** Tensiomyographic parameters of three skeletal muscles at hospital discharge (T_1_) and 2-month follow-up (T_2_).

	T_1_	T_2_	
	Mean (SD)	Mean (SD)	p_TIME_ (η^2^)
Vastus lateralis			
Delay time (ms)	24.6 (2.0)	23.6 (2.6)	.014 (.150)
Contraction time (ms)	31.3 (6.3)	26.9 (5.2)	<.001 (.358)
Amplitude (mm)	4.8 (2.1)	5.0 (1.8)	.547
Radial velocity (mm/ms)	.088 (.041)	.099 (.036)	.080
Biceps femoris			
Delay time (ms)	47.4 (10.2)	43.4 (11.1)	.091
Contraction time (ms)	29.8 (4.1)	27.8 (3.8)	.007 (.178)
Amplitude (mm)	5.3 (3.1)	6.4 (2.9)	.058
Radial velocity (mm/ms)	.072 (.045)	.091 (.040)	.016 (.144)
Gastrocnemius medialis			
Delay time (ms)	35.6 (8.3)	30.4 (8.3)	<.001 (.381)
Contraction time (ms)	25.1 (3.1)	23.1 (2.1)	<.001 (.313)
Amplitude (mm)	4.8 (1.6)	4.6 (1.8)	.459
Radial velocity (mm/ms)	.080 (.029)	.086 (.029)	.274

On average, participants rated their physical condition 18.1 percentage points higher after self-rehabilitation (at T_2_) than after hospitalization (at T_1_) (Figure 1), but the difference was not statistically significant (*p* = .078). A total of 25 out of 28 patients answered this question in the affirmative, reaching the pre-infection level of 100% (while three patients reported that their physical condition had further deteriorated at the 2-month follow-up compared to the pre-infection period).

However, the mental state assessment index remained almost unchanged. Participants rated their mental state 6.5 percentage points higher at T_2_ than at T_1_. Although most patients returned to normal, five out of 28 patients still felt that they had not returned to their pre-infection state (Figure 2).

### 3.1. Monitoring symptoms and changes in well-being during self-rehabilitation

During the two-month self-rehabilitation period, patients were asked to record any symptoms and changes in well-being. Only two patients returned a completed form with symptoms recorded. In addition, we were able to collect recalls of symptoms from 18 other patients at follow-up. Thus, in total, we recorded symptoms and changes in well-being in half of the patients who participated in the study (20 out of 40 participants) (Figure 3). Our data also show that the most common symptoms after COVID-19 are joint pain (10 patients), muscle pain, and cough (seven patients), followed by irritability, sadness, shortness of breath, and difficulty sleeping.

## 4. Discussion

In this study, we were interested in the clinical, functional, and cognitive status of patients recovering from hospitalization for COVID-19 who were not enrolled in an organized rehabilitation program. Measurements of their performance were taken immediately after hospital discharge and 2 months later. We found differences between T_1_ and T_2_ in BMI, fat-free mass, frailty score, muscle contractile properties, SARC-CalF, and MoCA scores.

### 4.1. Lifestyle characteristics and quality of life

We were interested in whether participants changed various lifestyle characteristics between T_1_ and T_2_. Nutritional and physical activity patterns should be changed after the COVID-19 hospital discharged to overcome negative consequences of disease. The self-reported amount of physical activity during self-rehabilitation period, on average, 5.2 h peer day 5.4 h per day of sitting. It has been suggested that COVID-19 may result in physical inactivity, which in turn may become a long-lasting symptom for individuals who have recovered from the virus (42). On the other hand, regular physical activity can have an important impact on different aspects of health, from cardiovascular to mental health (43). Another behavioral factor affecting COVID-19 rehabilitation is nutrition. Adequate nutrition can play a protective and regenerative role, according to COVID-19 (44). Because of its protective and preventive effects, it has been suggested that the Mediterranean diet may represent a positive approach against COVID-19 (45, 46). In our sample, we did not observe any changes in eating habits or lifestyle in favor of a Mediterranean diet between T_1_ and T_2_. Compared with other studies (47), our population had a comparable adherence to the Mediterranean lifestyle.

The Edmonton Frail scale and SARC-CalF test questionnaires were used to measure changes related to frailty or sarcopenia (27, 29). COVID-19 patients tend to be more frail after hospital discharge (48). Moreover, early detection of frailty and sarcopenia may help in the future planning of specific rehabilitation. We were interested in the frailty and sarcopenia status of the enrolled participants after COVID-19. At T_1_ measurements, two patients had severe frailty according to the Edmonton scale of frailty, three patients had a moderate frailty score, nine patients had a predisposition to frailty, and 25 patients showed no signs of frailty. The SARC-CalF test for sarcopenia showed that one patient had potential sarcopenia on T_1_ measurements. Two months after discharge, the frailty results improved, and no patient had severe frailty, while five patients exhibited signs of moderate frailty, and the remaining patients were not at risk for frailty. The SARC-CalF test for sarcopenia after two months showed that the patient no longer reported problems suggestive of sarcopenia. Therefore, the screening instruments (Edmonton Frail scale and SARC-CalF test), which are based on self-reporting and self-assessment, showed improvement in various aspects such as strength, assistance in walking, general health status, and functional independence.

### 4.2. Physical health and performance tests

Despite positive results at T_2_ for the Edmonton Frail scale and the SARC-CalF test, we did not find improvements in physical performance tests. As previously described in the literature, patients may have impaired physical performance after hospital discharge (49). Re-evaluation after two months of self-rehabilitation showed that participants did not achieve the results expected for a healthy population in selected functional parameters such as the 30-sec Sit-to-stand test, TUG, gait speed, grip strength, and clinical parameters such as systolic and diastolic blood pressure and FEV1/FVC. Due to the complexity of rehabilitation after COVID-19, bed exercises can be an effective tool in the initial stages. An example of bed exercises is the Full-body in-Bed Gym program (50, 51), which allows the difficulty of the exercises to be adjusted to the individual's physical and mental capabilities. Thermal water rehabilitation is also a possible suggestion for an effective rehabilitation program tailored to the individual. This type of rehabilitation can improve the inspiratory muscles, which are typically weakened after COVID-19 (52).

Patients had negatively deviated levels of some risk factors compared with recommended levels shortly after discharge. Factors such as increased BMI (53, 54) and higher systolic blood pressure (55, 56) have been previously identified as factors that may contribute to complications of COVID-19. In addition, it has also been suggested that COVID-19 increases systolic and diastolic blood pressure and may cause new-onset hypertension (57), but we did not find correlations between previously reported hypertension and elevated systolic blood pressure in our patients. Therefore, we can assume that the elevated systolic pressure in our patients is somehow related to the COVID-19 consequences.

#### 4.2.1. Skeletal muscle contractile properties

A very consistent change after self-rehabilitation (T_2_) was a decrease in temporal contractile parameters in three selected muscles, when compared to hospital discharge values (T_1_). Specifically, Tc decreased in all three muscles and Td decreased in VL and GM, while BF showed an almost significant decrease. Dm, which is related to muscle atrophy (58), remained unchanged. Both Td and Tc were found to be related to muscle MHC-I composition in VL muscle (25). This suggests a change toward fast-twitch fibers in VL at T_2_ when compared to T_1_. Although this is true for the VL muscle, there is no reason to believe that this would be similar in the other two observed muscles (GM and BF). In fact, our team previously reported no decrease in MHC VL proportions after 14 days of bed rest and recovery in healthy 55–65-year-olds (59, 60). Furthermore, the above-mentioned studies confirmed CSA, force, and specific force loss in only type I muscle fibers. Therefore, the explanation of shorter Td and Tc must be sought at lower activation levels, which are regularly found after short-term immobilization studies (61).

### 4.3. Psycho-sociological characteristics and subjective perceptions of illness

The literature also suggests changes in mental health status after hospitalization for COVID-19. Despite the anxiety and fear the respondents have experienced and, above all, the desire to regain their pre-infection health status shown on the first measurements at discharge from hospital care, two-thirds of the patients showed no significant improvement in the measured psychological parameters. At the T_1_ measurements, like Brown et al. (62), we noted a willingness to try anything to address symptoms after COVID-19, as subjects showed interest in exercising with the manual and in monitoring their health. Although they reported a severe experience with COVID-19, in addition to anxiety and health concerns, their lifestyle habits did not change after they went home, i.e., no systematic self-rehabilitation was reported at the T2 measurements.

The subjects' self-assessment of physical and mental status (index) showed mainly an improvement in physical status, where the assessment of mental status remained unchanged, while some patients (5%) still didn't reach the pre-Covid state, which may indicate that the mental (mood) consequences are lasting longer what confirmed also by the MoCA test. Despite an average higher MoCA score in the whole sample, 15 out of 39 patients had worsened or remained unchanged at 2 months. Nevertheless, patients' adherence appeared to be very low, as they admitted that they did not follow the manual as advised at the initial measurements. How to encourage patients to take a more active approach to rehabilitation and how to increase adherence will certainly be our future challenges.

Improvements in the MoCA test are indicative of recovery of cognitive function in hospitalized COVID-19 patients at 2 months after hospital discharge. The original MoCA validation study reported a test-retest consistency of 0.91 at 2 months, with no significant learning effect (63); however, the indication of improved performance following repeated MoCA administrations (64) stresses the importance of interpreting the results with caution. It has already been established that patients who are critically ill or who have been treated in an intensive care unit (ICU) are at risk of suffering from the long-term consequences of COVID-19, such as impaired physical and cognitive function and psychological disorders similar to post-intensive care syndrome (PICS). Therefore, optimally selected rehabilitation may be required to address these disabilities (65). In addition to the significance of the results indicating overall recovery, it is important not to overlook the 15 (38.5%) participants whose cognitive status either worsened or remained unchanged from post-hospitalization to 2-month follow-up. These participants may be at higher risk or more susceptible to long-term post-infection difficulties and may require additional attention during recovery, if successful. A comprehensive report on the cognitive status of the patients enrolled in this study has been published previously (66).

### 4.4. Limitations

The single-center design and relatively small number of patients are potential limitations of this study and may limit the generalizability of the results. The severity of the infection was not considered, and no assessment was performed before or during the infection, so changes may not be solely attributable to the infection.

## 5. Conclusions

COVID-19 has long-term negative consequences regardless of the stage of the disease (67), making it all the more important that patients have the opportunity for organized rehabilitation. Although the subjects were provided with materials for self-rehabilitation and entered the recovery phase with desire and enthusiasm, this was not sufficient to engage in regular physical activity that would constitute post-disease rehabilitation. This was also reflected in the tests, as there was no visible improvement in the physical performance tests. Our results confirm that patients do not recover all functions within two months without organized or guided rehabilitation. Rehabilitation treatment for post-COVID-19 patients discharged from the hospital should be tailored to each individual's needs. This includes recovery from muscular and neurological deficits, cardiorespiratory reconditioning, improvement of cognitive symptoms, and education on healthy lifestyles. Therefore, it is important to provide patients with organized and guided complex rehabilitation that includes clinical, cognitive, and motor exercises.

## Data Availability

The raw data supporting the conclusions of this article will be made available by the authors, without undue reservation.
